# Exosomal miRNA Biomarker Panel for Pancreatic Ductal Adenocarcinoma Detection in Patient Plasma: A Pilot Study

**DOI:** 10.3390/ijms24065081

**Published:** 2023-03-07

**Authors:** Amy Makler, Waseem Asghar

**Affiliations:** 1 Micro and Nanotechnology in Medicine, Department of Electrical Engineering and Computer Science, College of Engineering and Science, Florida Atlantic University, Boca Raton, FL 33431, USA; 2 Department of Biomedical Science, Charles E. Schmidt College of Medicine, Florida Atlantic University, Boca Raton, FL 33431, USA

**Keywords:** biomarker panel, diagnostics, exosomes, microRNA, pancreatic ducal adenocarcinoma, plasma

## Abstract

Pancreatic ductal adenocarcinoma (PDAC) is rapidly becoming one of the leading causes of cancer-related deaths in the United States, and with its high mortality rate, there is a pressing need to develop sensitive and robust methods for detection. Exosomal biomarker panels provide a promising avenue for PDAC screening since exosomes are highly stable and easily harvested from body fluids. PDAC-associated miRNAs packaged within these exosomes could be used as diagnostic markers. We analyzed a series of 18 candidate miRNAs via RT-qPCR to identify the differentially expressed miRNAs (*p* < 0.05, *t*-test) between plasma exosomes harvested from PDAC patients and control patients. From this analysis, we propose a four-marker panel consisting of miR-93-5p, miR-339-3p, miR-425-5p, and miR-425-3p with an area under the curve (AUC) of the receiver operator characteristic curve (ROC) of 0.885 with a sensitivity of 80% and a specificity of 94.7%, which is comparable to the CA19-9 standard PDAC marker diagnostic.

## 1. Introduction

Pancreatic ductal adenocarcinoma (PDAC) is the third leading cause of cancer-related deaths in the United States of America with an estimated 62,000 new diagnoses and an estimated 50,000 deaths expected in the USA this year [1]. Currently, the only FDA-approved diagnostic for PDAC is serum antigen CA19-9. Patients with cancer of the pancreas, stomach, lung, liver, or colon typically show levels of CA19-9 exceeding 37 U/mL [2]. For PDAC detection, the CA19-9 serum antigen diagnostic test has a sensitivity ranging between 79% and 95% and a specificity ranging between 82% and 91% [3]. However, since non-cancerous conditions such as pancreatitis, gallbladder infection, liver disease, and gallstones may also show increased CA19-9 levels [4,5], there is a need to develop new modalities with increased sensitivity and specificity of detection for PDAC compared to non-cancerous conditions.

Exosomes, and their contents, may offer a more reliable diagnostic alternative to CA19-9. Exosomes are released by all cells in the body, and it is well established that tumor cells release even greater quantities of exosomes [6]. Exosomes are 30–150 nm sized extracellular vesicles that contain proteins, DNA, RNA, and other cellular constituents [7,8]. They are stable in body fluids, allowing for easy collection from patient blood, plasma, serum, saliva, or urine. Recent research has examined both exosomes and their contents for diagnostic feasibility for many diseases [7,8,9,10,11]. 

The microRNA transcriptome potentially contains diagnostic biomarkers for PDAC that could exceed the sensitivity and specificity of CA19-9 serum markers. MicroRNAs are 19–25-nucleotide-long sequences that have been shown to regulate about a third of human genes, with half being involved in tumor regulation [12]. In non-cancerous cells, miRNAs play roles in a variety of metabolic processes including embryogenesis, growth, repair, cell cycle, proliferation, stress tolerance, and immune response [13,14]. In cancerous tumors, they can play roles in drug resistance, immune evasion, growth, and metastasis. MicroRNAs (miRNAs) can exhibit tumor suppressor or oncogenic roles, with some miRNAs exhibiting both, depending on tissue and tumor type. Functional analyses have examined the roles of miRNA in PDAC progression. For example, miR-196b was implicated in driving PDAC progression by interacting with known PDAC-associated miR-21 and miR-31 [15]. Inhibition of miR-196b resulted in decreased levels of miR-21 and miR-31 as well as a decrease in cell proliferation. Because of these tissue-specific roles, miRNAs associated with abnormalities in cellular metabolic processes characteristic of specific tumors could be used as a more sensitive and specific diagnostic method. 

Circulating miRNAs and miRNAs in various body fluids have been extensively researched with promising results. In lung cancer, multiple panels of microRNAs isolated from peripheral blood were used to diagnose early lung cancer compared to the control [16]. Several studies have reported utilizing urinary, plasma, and serum miRNAs to detect bladder cancer [17]. Similarly, there have been several studies that examine circulating miRNAs and miRNAs in body fluids for diagnosis, prognosis, and monitoring response to therapy. Liu et al. reported a serum marker comprised of seven miRNAs that could distinguish PDAC from chronic pancreatitis with 83.6% accuracy [18]. Another study analyzed plasma from PDAC patients and control patients and found miR-21 and miR-483-3p to be significantly increased in PDAC compared to the control [19]. Additional miRs and their roles in PDAC diagnosis have been reported and summarized previously [7,20,21].

Dysregulation of miRNAs and exosomes in cancer have been shown to offer highly sensitive and specific methods for diagnosing respective tumor types, including PDAC [7,8,22]. In our previous study, we developed a workflow strategy to identify a panel of miRNAs for potential pancreatic ductal adenocarcinoma (PDAC) detection [23]. Multiple knowledgebases were accessed to generate a database of 383 PDAC-associated non-coding RNAs (ncRNAs), with the majority belonging to the miRNA subtype. The cBioPortal [24] tool was used to identify 72 miRNAs that exhibited alteration in at least 10% of the University of Texas Southwestern (UTSW) PDAC dataset (N = 109). These 72 miRNAs were enriched for their presence in exosomes, resulting in 50 exosomal miRNA. The cBioPortal batch analysis function was used to test combinations of these exosomal miRNA and identified a final panel of 18 mature miRNAs that exhibited alteration in 90% of the UTSW PDAC dataset. These MIRs provided the basis for the design of a diagnostic panel with the potential for early detection and monitoring of PDAC. These 18 were then analyzed in vitro to provide the basis for testing in plasma derived from PDAC and control patients. In the present study, we used quantitative RT-PCR to measure the levels of these 18 mature miRNA from exosomes harvested from PDAC patient plasma compared to control patient plasma. Four of these 18 candidate exosomal miRNAs exhibited significant expression differences between control and PDAC patient plasma samples. The diagnostic potential of these four miRs was explored and found to be comparable to CA19-9 in sensitivity and specificity, but they exhibited greater potential for detecting early stage PDAC compared to CA19-9.

## 2. Results

### 2.1. Patient Information

In our previous study, we analyzed the expression levels of 18 candidate mature miRNAs extracted from exosomes released from in vitro cultured PDAC cell lines versus an immortalized pancreatic cell line [23]. Seven of the 18 candidate mature miRNAs were found to be differentially expressed between the experimental and control groups, suggesting these exosomal miRNAs could potentially be used as a diagnostic panel for PDAC. We sought to design a diagnostic panel for PDAC based on the expression of these same 18 candidate exosomal miRNAs and their differential expression in plasma collected from PDAC patients (N = 15) and control patients (N = 19). Sex, age, ethnicity, CA19-9 levels, and tumor staging are reported in Table 1. Seven of the 15 PDAC patients exhibited CA19-9 levels within normal ranges (<37 U/mL). 

### 2.2. RT-qPCR Analysis of Exosomal miRNAs from PDAC Patient Plasma

Exosomal miRNAs were isolated from plasma collected from PDAC and control patients. Expression levels of 18 candidate miRNAs were measured using RT-qPCR. After 40 cycles of PCR, 7 of the 18 candidate miRNAs were not detectable in over 80% of the samples (N = 34) and, thus, were excluded from further analysis as recommended by a previous study [23]. The remaining 11 mature miRNAs (miR-93-5p, miR-93-3p, miR-133a-3p, miR-210-3p, miR-330-5p, miR-330-3p, miR-339-5p, miR-339-3p, miR-425-5p, miR-425-3p, and miR-3620-3p) were further analyzed for differential expression between PDAC and control patients using ΔCq values.

**Figure 1:**
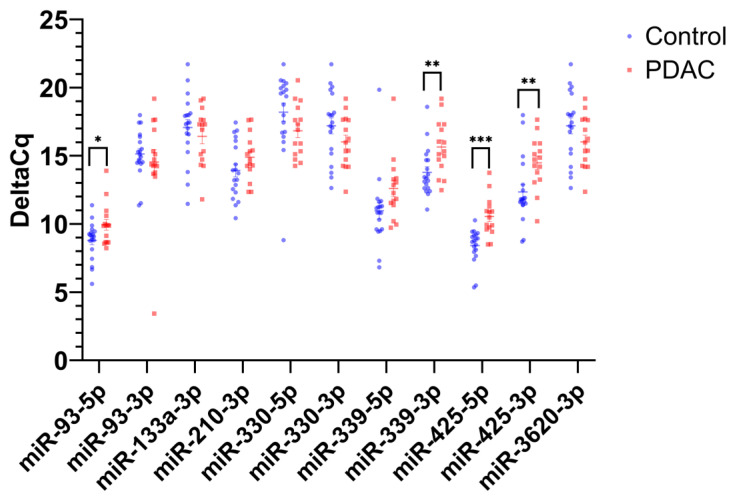
Exosomal miRNA expression levels in PDAC patient plasma compared to control. The scatterplot shows the average ΔCq values for 11 mature miRNAs in PDAC samples (N = 15) and control samples (N = 19). Student’s *t*-test was used to establish significance, where *, **, and *** denote *p* < 0.05, *p* < 0.01, and *p* < 0.001, respectively.

RT-qPCR analysis of exosomal miRNA identified four mature miRNAs that exhibited statistically significant expression differences between the control and PDAC patient plasma samples: miR-93-5p (*p* < 0.05, 99% confidence interval (CI) control ΔCq range: 7.99–9.60 and 99% CI PDAC ΔCq range: 8.92–10.96), miR-339-3p (*p* < 0.01, 99% CI control ΔCq range: 12.75–14.82 and 99% CI PDAC ΔCq: 14.27–16.84), miR-425-5p (*p* < 0.001, 99% CI control ΔCq: 7.67–9.16, 99% CI PDAC ΔCq: 9.59–11.51), and miR-425-3p (*p* < 0.01, 99% CI control ΔCq: 10.93–13.77, 99% CI PDAC ΔCq: 13.15–15.64) (Figure 1). All four miRNAs exhibited significantly increased ΔCq values in PDAC samples compared to the control. These four miRNAs constituted the best candidate miRNAs for an exosomal miRNA PDAC diagnostic and were further analyzed for associated biological functions and pathways.

### 2.3. Gene Ontology and KEGG Pathway Analysis of Differentially Expressed Plasma Exosome miRNAs

We utilized the DIANA-miRPATH v3 tool [25] to identify biological pathways and functions of the four miRNAs (miR-93-5p, miR-339-3p, miR-425-3p, miR-425-5p) using the DIANA-TarBase 7.0 option. Kyoto Encyclopedia of Genes and Genomes (KEGG) [26] analysis revealed that miR-93-5p was involved in several cancer-specific pathways (glioma, bladder cancer, chronic myeloid leukemia, renal cell carcinoma, colorectal cancer, and pathways in cancer), while miR-93-5p and miR-425-5p were both involved in cancer-regulatory pathways including p53 signaling and HIPPO signaling pathways [27] (Supplementary Figure S1A). Gene ontology analysis revealed that shared functions of three of the four miRs (miR-93-5p, miR-339-3p, and miR-425-5p) included cellular nitrogen compound metabolic processes, gene expression, RNA binding, and protein metabolic processes (Supplementary Figure S1B).

### 2.4. PDAC Stage-Specific Differences in Plasma Exosome miRNA Expression Levels

**Figure 2:**
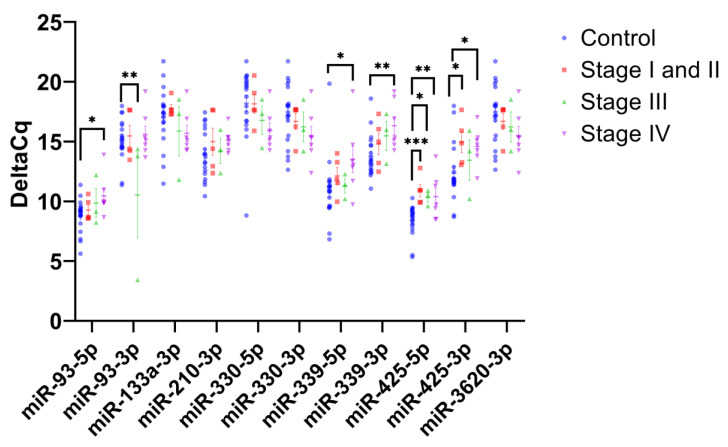
PDAC stage-specific expression levels of plasma exosomal miRNAs. The scatterplot shows the average ΔCq values for 11 mature miRNAs in PDAC samples separated by staging, early stage (stage I and II, N = 5), mid stage (stage III, N = 3) and late stage (stage IV, N = 7), and compared to control samples (N = 19). Student’s *t*-test was used to establish significance, where *, **, and *** denote *p* < 0.05, *p* < 0.01, and *p* < 0.001, respectively.

The RT-qPCR analysis identified four miRNAs (miR-93-5p, miR-339-3p, miR-425-5p, and miR-425-3p) with significantly higher ΔCq in plasma exosomes from PDAC patients compared to the control. PDAC samples were further divided into “Early stage” (stage I and II, N = 5), “Mid stage” (stage III, N= 3), and “Late stage” (stage IV, N = 7) to identify miRNAs with stage-specific differences in expression levels compared to control samples (Figure 2). Analysis of early-stage PDAC samples revealed that miR-425-5p and miR-425-3p had significantly higher ΔCq values compared to control samples (*p* < 0.001, and *p* < 0.05, respectively). Analysis of mid-stage PDAC samples revealed that miR-93-3p had a significantly lower ΔCq value compared to control samples (*p* < 0.01), suggesting greater expression, and miR-425-5p exhibited a significantly higher ΔCq value compared to the control (*p* < 0.05). An analysis of late-stage PDAC samples revealed that miR-93-5p, miR-339-5p, miR-339-3p, miR-425-5p, and miR-425-3p all had significantly higher ΔCq values compared to the control samples (*p* < 0.05, *p* < 0.05, *p* < 0.01, and *p* < 0.05, respectively). 

### 2.5. Diagnostic Value of Plasma Exosomal miRNAs

**Figure 3:**
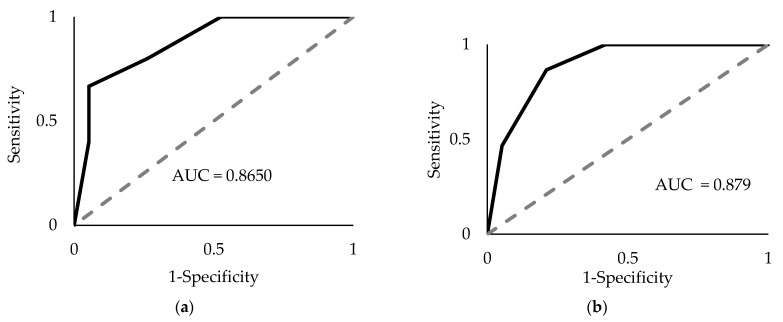
Receiver operator characteristic (ROC) area under the curve (AUC) analyses. The four-miRNA panel (miR-93-5p, miR-339-3p, miR-425-5p, and miR-425-3p) underwent ROC analysis using the upper limits of the average control ΔCq thresholds (**a**) and using the lower limit of the average PDAC ΔCq thresholds (**b**). The values are based off the average ΔCqs for each miRNA in either control or PDAC plasma samples with 99% CI for all such values.

**Figure 4:**
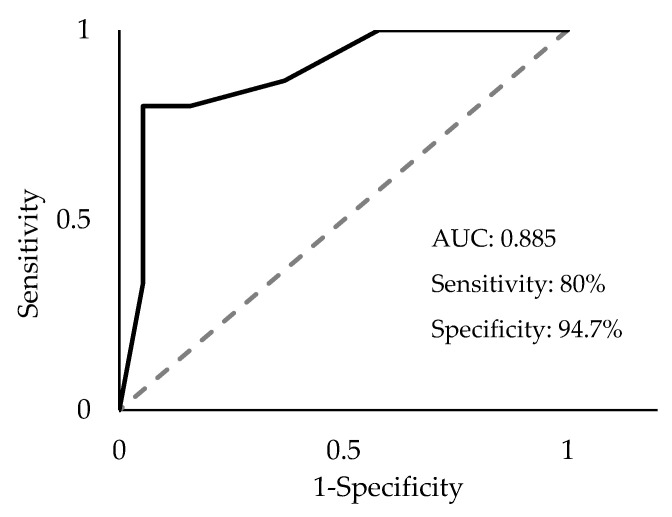
Receiver operator characteristic (ROC) area under the curve (AUC) analysis of the combined four-miRNA panel using two thresholds. Sensitivity = 80%, specificity = 94.7%; AUC = 0.885, CI 99% 0.74–1.00, *p* < 1 × 10^−10^.

**Figure 5:**
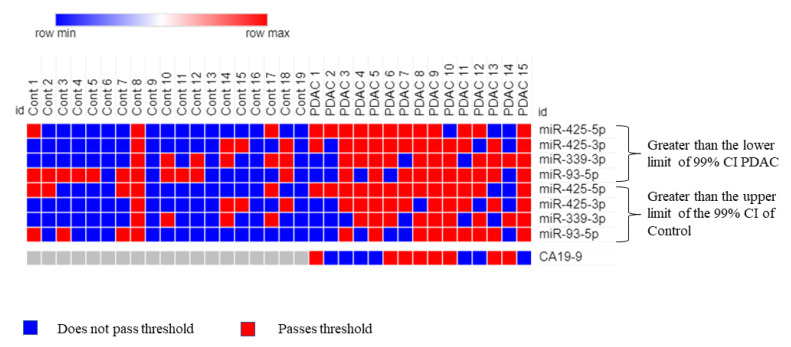
Analyzing PDAC and control samples using the combined four-miRNA biomarker panel. The heatmap shows which samples surpassed the two thresholds (ΔCq is greater than the upper limit of the control for each miRNA AND ΔCq is greater than the lower limit of PDAC for each miRNA, 99% CI), indicating a positive hit for PDAC. Red indicates a positive hit and blue indicates a negative hit. Columns labeled as “Cont” represent control plasma samples while columns labeled as “PDAC” represent plasma from patients with pancreatic cancer. PDAC 1, 2, 3, 4, and 5 represent early-stage (I and II) pancreatic cancer; PDAC 6, 7, and 12 represent mid-stage (stage III) pancreatic cancer; and PDAC 8, 9, 10, 11, 13, 14, and 15 represent late-stage (stage IV) pancreatic cancer. CA19-9 values are added for comparison where the threshold is the medically established value of >37 U/mL.

Receiver operative characteristic (ROC) curve analysis was performed on the four mature miRNAs to assess their combined diagnostic efficacy. Since all four miRNAs (miR-93-5p, miR-339-3p, miR-425-5p, and miR-425-3p) exhibited significantly greater ΔCq values in PDAC samples compared to the control, two threshold values were used to establish a positive “hit” for diagnosing PDAC. If a sample exhibited an miRNA with a ΔCq value that was (1) greater than the upper limit of the 99% confidence interval (CI) of the average control sample and (2) greater than the lower limit of the 99% CI of the average PDAC sample ΔCq value, it was recorded as a positive hit for PDAC. Additionally, if a sample exhibited an miRNA with a ΔCq value greater than both thresholds, it was recorded as two positive hits for PDAC. The upper limit, or the highest average ΔCq value for the control, was determined for each miRNA and used as a threshold for establishing a potential diagnostic. These values (99% CI) were >9.60 for miR-93-5p, >14.82 for miR-339-3p, >9.16 for miR-425-5p, and >13.77 for miR-425-3p. Using these thresholds, a panel consisting of these four miRNAs was assessed via ROC analysis. The AUC was 0.865 (*p* < 1 × 10^−8^, Figure 3a) with a sensitivity of 66.7% and a specificity of 94.7%. Similarly, the lower limit of the average PDAC ΔCq value (99% CI) for each miRNA as the threshold was >8.92 for miR-93-5p, >14.27 for miR-339-3p, >9.59 for miR-425-5p, and >13.15 for miR-425-3p. If the values exceeded the minimum average ΔCq for PDAC, it was considered a positive hit for PDAC detection. Using this threshold, the four-miRNA panel yielded an AUC of 0.878 (*p* < 1 × 10^−9^, Figure 3b), a sensitivity of 80%, and a specificity of 89.5%. Finally, combining both the upper limits of the control thresholds (99% CI) and the lower limits of the PDAC thresholds (99% CI), (the ROC analysis resulted in an AUC of 0.877 (*p* < 1 × 10^−9^), and an overall sensitivity of 80% and specificity of 94.7% (Figure 4). Therefore, this marker allows us to detect PDAC in 12 of the 15 samples, including four out of five early-stage PDAC patients. By comparison, CA19-9 could only detect PDAC in 8 out of the 15 samples and was only able to detect one out of the five early-stage PDAC patients (Figure 5). Interestingly, control sample 8 exhibited the maximum possible of 8 positive hits for PDAC (2 hits for each of the four miRNA), which marks it as a clear outlier sample compared to the other control samples (Figure 5). The provided patient history for the control samples was limited, and, therefore, it could not be verified whether or not control patient 8 may have had a known pancreatic cancer diagnosis. 

## 3. Discussion

Pancreatic cancer continues to be a difficult disease to diagnose and treat. Mortality rates for PDAC are high, though each year has brought marginal improvements in survival rate, with 2022 reporting a survival rate of 11% [1]. The need to develop more reliable methods of detecting pancreatic cancer at earlier stages remains a top priority. Some avenues of research have turned to freely circulating miRNAs or exosomal miRNAs for an array of diseases, including various cancers [28]. Recently, a study by Zou et al. proposed a panel for the early detection of PDAC comprising six circulating miRNAs (let-7b-5p, miR-192-5p, miR-19a-3p, miR-19b-3p, miR-223-3p, and miR-25-3p) that were identified by machine learning and validated in patient samples [29]. Another study by Wang et al. discovered a single serum exosomal marker, miR-1226-3p, that could diagnose and predict pancreatic cancer invasion and metastases [30]. Despite these important contributions, there remain alternative diagnostic biomarkers to be discovered with the potential for earlier and more reliable detection of pancreatic cancer. 

Our previous study identified 18 candidate miRNAs from a bioinformatics analysis of publicly available cBioPortal data collected from pancreatic cancer patients [23]. Seven miRNAs were verified to be differentially expressed in exosomes collected from PDAC cell lines compared to an immortalized pancreatic cell line in vitro. Therefore, we sought to validate the expression levels of these candidate miRNAs in plasma exosomes collected from known PDAC patients. The present study identified four miRNAs, miR-93-5p, miR-339-3p, miR-425-5p, and miR-425-3p, with significantly greater ΔCq values in PDAC plasma samples compared to control plasma samples. A biomarker panel consisting of these four miRNAs and a dual threshold cutoff consisting of the upper limit of the ΔCq values (99% CI) of the miRNAs from control samples and the lower limit of the ΔCq values of the miRNAs (99% CI) from PDAC samples resulted in a diagnostic with an AUC of 0.887, a sensitivity of 80%, and specificity of 94.7%. This is comparable to the current FDA-approved CA19-9 diagnostic, which exhibits both a variable sensitivity (70–90%) and specificity (68–91%) [31]. Interestingly, CA19-9 levels were reported as elevated in only 8 of the 15 (53.33%) PDAC patient samples collected for this study. For comparison, by using the optimal cutoff of the four-miRNA two-threshold biomarker panel proposed in this study, our panel positively identified 12 of the 15 (80.00%) PDAC patient samples. This could indicate a potential improvement in PDAC diagnostic sensitivity using the proposed biomarker panel compared to the established CA19-9 serum diagnostic. However, further studies on a larger sample size are needed to fully evaluate this novel finding. 

Our study also identified that both mature forms of miR-425 (miR-425-5p and miR-425-3p) had significantly greater ΔCq values in stage I and II PDAC compared to controls. Consistently, when we applied the two-threshold diagnostic cutoff solely to miR-425-5p, we were able to positively identify five out of five (100% sensitivity) early-stage pancreatic cancer samples. By comparison, CA19-9 was only able to detect one out of five (20%) early-stage pancreatic cancer samples. Therefore, miR-425-5p may offer a more sensitive diagnostic for early-stage PDAC compared to the FDA-approved CA19-9.

Four miRNAs had significantly lower levels of expression in plasma exosomes extracted from PDAC patients compared to control patients. Some of these miRNAs have been previously associated with a variety of cancers and have been implicated in tumor development. For example, miR-93-5p appears to play a role in tumor suppression in ovarian [32] and breast cancers [33,34] by targeting the PD-L1/CCND1 pathway, which is involved in regulating the cell cycle. This is in contrast to the MIR-93 pre-transcript and miR-93-3p, which exhibit oncogenicity. MIR-93 is associated with poor prognosis in PDAC [35], while miR-93-3p predicts poorer outcomes in patients with triple-negative breast cancer [36]. Similarly, overexpression of miR-93-5p appears to promote chemoresistance in PDAC by targeting the PTEN/PI3K/Akt signaling pathway, which is typically involved in tumor suppression [37]. Additional studies confirm the pro-tumorigenic activities of miR-93-5p in endometrial and PDAC tumors [37,38,39,40]. Interestingly, our data are consistent with the previous studies for miR-93-3p, which show a decrease in ΔCq values, suggesting an increased expression as PDAC transitions from early to late stages. MicroRNA-425-3p is known to be upregulated in response to cisplatin treatment in non-small cell lung carcinoma (NSCLC) tissue and exosomes [41]. MicroRNA-425-5p has been shown to be expressed at higher levels in PDAC tissue compared to adjacent healthy tissue [42] as well as in NSCLC [43] and in the serum of gastric cancer patients [44]. Exosomal miR-425-5p from MDA-MB-23 breast cancer cells was shown to convert normal fibroblasts to cancer-associated fibroblasts upon uptake by suppressing the expression of TGFβRII, a TGFβ receptor [45]. Additional research has reported that miR-425-5p promotes tumorigenesis in colorectal cancer by inhibiting the PTEN-p53/TGFβ axis [46] and by activating the CTNND1-mediated β-catenin pathway [47]. MicroRNA-339-5p has been shown to suppress colorectal cancer progression by targeting PRL-1 [48], and while miR-339-3p was observed to interfere with CRC progression, its mechanism is unknown [49]. MiR-339-5p has also been implicated in suppressing melanoma by targeting MCL1, which promotes chemoresistance [50]. Little is known about the role miR-339-3p plays in PDAC, though one paper reports that it is downregulated in the PDAC cell line MIA PaCa-2 [51]. Another work implicates miR-339-3p in inhibiting caerulin-induced acute pancreatitis by targeting TRAF3, which promotes inflammation in pancreatitis cells [52]. Although these previous studies indicate potential roles for miR-93-5p, miR-339-3p, miR-425-5p, and miR-425-3p in cancers, to our knowledge, the present study is the first to propose a diagnostic panel for PDAC using all four biomarkers in plasma exosomes.

Although the present findings indicate a potentially promising novel diagnostic panel to detect pancreatic cancer from plasma exosomes, we cannot rule out the risk of a type I error and the chance that the results are a false positive due to a small sample size. Additionally, a history of cancer treatment regimens for each of the PDAC patients was not available. It is possible that some of the observed miRNA expression changes are due to responses to cancer treatment therapies rather than due to cancer progression. Nevertheless, the proposed four-miRNA two-threshold diagnostic biomarker panel was found to perform comparably to the established CA19-9 diagnostic marker. Further studies on a larger sample size would need to be conducted to conclusively evaluate the diagnostic performance of the proposed panel. To rule out the possibility of sex-, age-, and ethnicity-specific differences, chi-squared tests were performed between the control and PDAC patient information. Chi-squared analyses revealed no significant differences for sex (*p* > 0.05) and ethnicity (*p* > 0.05); however, there was a significant difference in the age (*p* < 0.05) of the PDAC group compared to the control group, when all ages are factored in. Our control group comprised 9 age-matched individuals and 11 individuals who were under the age of 40. There is no significant difference in age variation (*p* > 0.05), however, between the nine age-matched control samples (Cont 2 and Cont 9–16, Figure 4) and their PDAC counterparts. In fact, our data show a clear difference in the expression patterns of the miRNAs (miR-93-5p, miR-339-3p, miR-425-5p, and miR-425-3p) between our age-matched control and PDAC samples. However, the sample set remains small, and further studies will require a larger sample size with more closely matched sample demographics.

In summary, the proposed four-miRNA biomarker panel is able to accurately diagnose PDAC (sensitivity = 80%) compared to control (specificity = 94.7%) samples, performs comparably, and is potentially superior to the established CA19-9 diagnostic method. Additionally, miR-425-5p was identified as a potential marker for the early detection of pancreatic cancer at stages I and II. Further investigation is required to fully evaluate this panel for PDAC diagnosis and monitoring.

## 4. Materials and Methods

### 4.1. Sample Collection

Plasma samples from 15 patients (male: 13, mean age: 66.54; female: 2, mean age: 70) diagnosed with pancreatic cancer were provided by Baptist Health South Florida. The pancreatic cancer samples comprised 10 previously banked plasma specimens collected between 2018 and 2020 and 5 plasma specimens collected in December 2021 by Baptist Health South Florida Hospital. Sex, age, race, staging, and CA19-9 levels are reported in Table 1. Whole blood samples from 19 control patients (male: 13, mean age: 42.62; female: 6, mean age: 44.17) were provided by Continental Blood Bank, Ft. Lauderdale, FL, between 2020 and 2021. The control samples were tested to ensure they were clean of standard blood-borne pathogens prior to release by the blood bank. Patient age, sex, and race are reported in Table 1. In-depth medical history was not recorded by the provider. Plasma was separated from whole blood by centrifugation for 10 min at 2000 g. All samples were deidentified prior to acquisition and stored at −80 °C until exosome isolation steps.

### 4.2. Exosomal miRNA Isolation

Exosomes were isolated from plasma samples using the PEG-based Total Exosome Isolation Kit (from plasma) (Invitrogen, Waltham, MA, USA) as per manufacturer instructions. The Total RNA and Protein Isolation kit (Invitrogen, Waltham, MA, USA) was utilized for the extraction of miRNA from the exosomes isolated from plasma samples. MicroRNA was extracted as per manufacturer instructions, with the addition of an exogenous spike-in control of 1.5 pg of miR-cel-2-3p (Applied Biosystems, Foster City, CA, USA) to monitor RNA extraction efficiency.

### 4.3. RT-qPCR Analysis

The TaqMan™ Advanced miRNA cDNA Synthesis Kit (Applied Biosystems, Foster City, CA, USA) and TaqMan™ Fast Advanced Master Mix Kit (Applied Biosystems, Foster City, CA, USA) was used to prepare the miRNA for qPCR. The following TaqMan™ Advanced miRNA Assays (Applied Biosystems, Foster City, CA, USA) were used: cel-miR-2-3p (Assay ID: 478291_mir), miR-16-5p (Assay ID: 477860_mir), miR-31-3p (Assay ID: 478012_mir), miR-31-5p (Assay ID: 478015_mir), miR-93-3p (Assay ID: 478209_mir), miR-93-5p (Assay ID: 478210_mir), miR-133a-3p (Assay ID: 478511_mir), miR-133a-5p (Assay ID: 478706_mir), miR-210-3p (Assay ID: 477970_mir), miR-210-5p (Assay ID: 478765_mir), miR-330-3p (Assay ID: 478030_mir), miR-330-5p (Assay ID: 478830_mir), miR-339-3p (Assay ID: 478325_mir), miR-339-5p (Assay ID: 478040_mir), miR-425-3p (Assay ID: 478093_mir) miR-425-5p (Assay ID: 478094_mir), miR-429 (Assay ID: 477849_mir), miR-1208 (Assay ID: 478637_mir), miR-3620-3p (Assay ID: 479690_mir), and miR-3620-5p (Assay ID: 480850_mir).

RT-qPCR was performed using the AriaMX Thermocycler (Agilent, Santa Clara, CA, USA). The PCR settings are described in TaqMan™ Fast Advanced miRNA cDNA Synthesis Kitprotocols. To normalize sample Cq values, the exogenous spiked-in cel-miR-2-3p control and endogenous hsa-miR-16-5p control Cq values were averaged for each sample. All samples were run in duplicate. MicroRNA levels were calculated and expressed as ΔCq between the control miRNA Cq value (average of cel-miR-2-3p and hsa-miR-16-5p) and each of the 11 candidate miRNA Cq values. Due to variability in the initial volume of the plasma samples provided, a Cq adjustment was performed to normalize all samples to 750 uL by using the following formula:

$$Cq_{norm}=Cq_{raw}-\log_{2}\left(\frac{750\;uL}{X}\right)$$

where *X* = Initial sample volume in uL.

To minimize PCR background effects, miRNA with Cq values over 35 or not detected after 40 cycles was adjusted to a Cq value of 36 to test for differential expression between PDAC samples versus control samples. The full protocol has been described elsewhere [53].

### 4.4. Statistical Validation

Student’s two-tailed *t*-test was used to test for statistically significant differences in exosomal miRNA expression levels between control and PDAC samples. The chi-squared test was used to predict the associations between age, sex, or ethnicity in the control vs. PDAC groups.

## Figures and Tables

**Table 1:** Patient information obtained from 19 control donors and 15 pancreatic cancer donors.

	Control (N = 19)	Pancreatic Cancer (N = 15)
Sex N (%)		
Male	13 (68.4%)	13 (86.7%)
Female	6 (26.3%)	2 (13.3%)
Mean Age (Range) in years		
Male	42.62 (23–67)	66.54 (40–81)
Female	44.17 (21–67)	70 (62–78)
Ethnicity N (%)		
Caucasian	1 (5%)	3 (20%)
African American	7 (40%)	1 (6.7%)
Hispanic	9 (45%)	11 (66.7%)
Non-white Hispanic	2 (10%)	
CA19-9		Normal: 7 (47%) Elevated: 8 (53%)
Tumor Stage		
I		3 (20%)
II		2 (13.3%)
III		3 (20%)
IV		7 (46.7%)

## Data Availability

The data are within the article and Supplementary Files.

## Supplementary Materials

The supporting information can be downloaded at: https://www.mdpi.com/article/10.3390/ijms24065081/s1.
