# Supplementary material for: Exosomal miRNA Biomarker Panel for Pancreatic Ductal Adenocarcinoma Detection in Patient Plasma: A Pilot Study
Source: Int J Mol Sci. 2023 Mar 7;24(6):5081. doi: 10.3390/ijms24065081 (PMC10049393; doi:10.3390/ijms24065081)
Supplement: Supplementary file 1 [file ijms-24-05081-s001.zip › Supplementary Figure S1Legend.pdf]

Supplementary Figure S1. KEGG pathways and Gene ontology analysis of differentially expressed plasma exosome miRNAs. Heatmaps show the top most statistically significant KEGG pathways (A) and Gene Ontology terms (B) associated with the four miRs (miR-425-5p, miR-425-3p, and miR-339-3p, miR-93-5p).
