# Supplementary figures and images for: Exosomal miRNA Biomarker Panel for Pancreatic Ductal Adenocarcinoma Detection in Patient Plasma: A Pilot Study
Source: Int J Mol Sci. 2023 Mar 7;24(6):5081. doi: 10.3390/ijms24065081 (PMC10049393; doi:10.3390/ijms24065081)

A

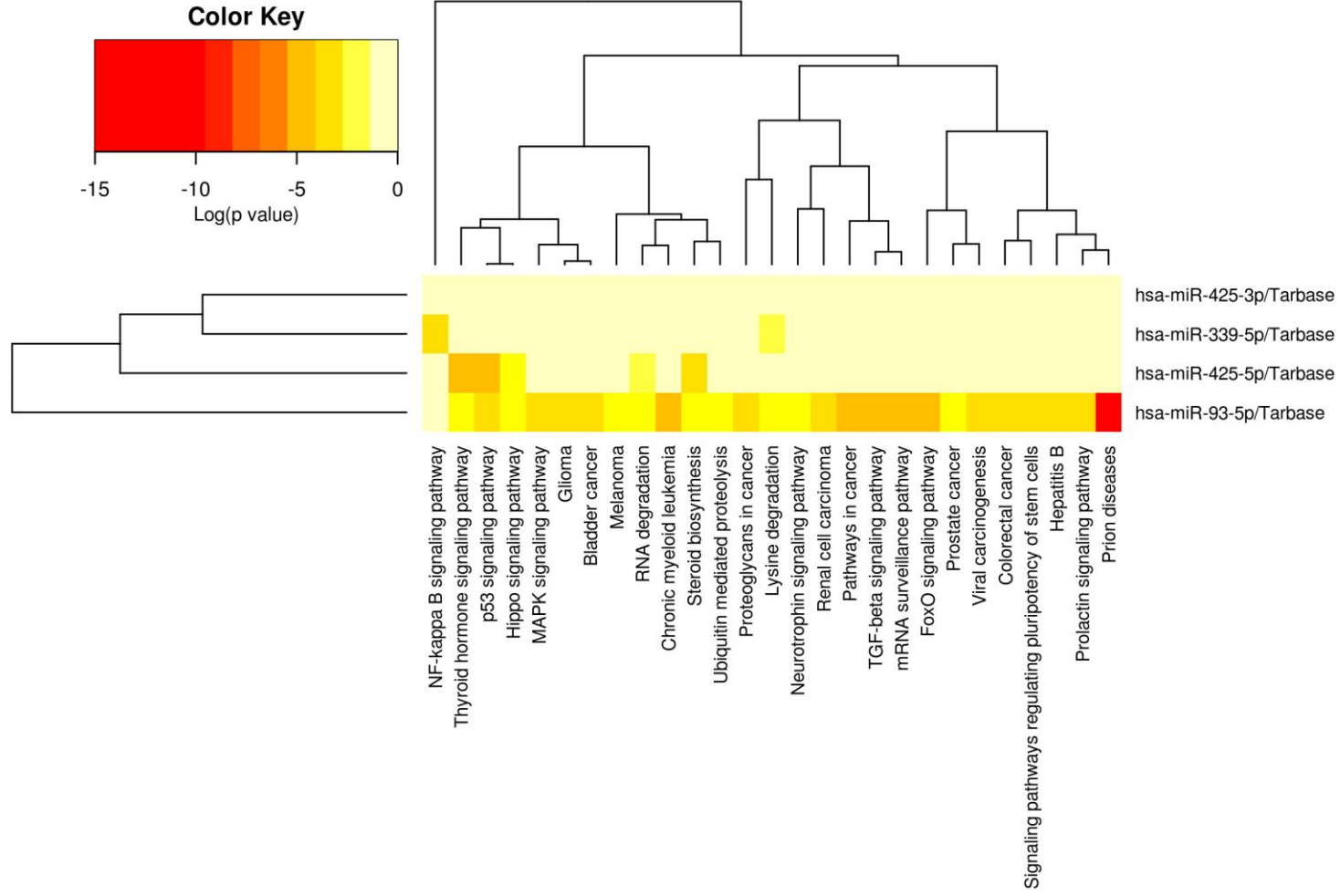

B

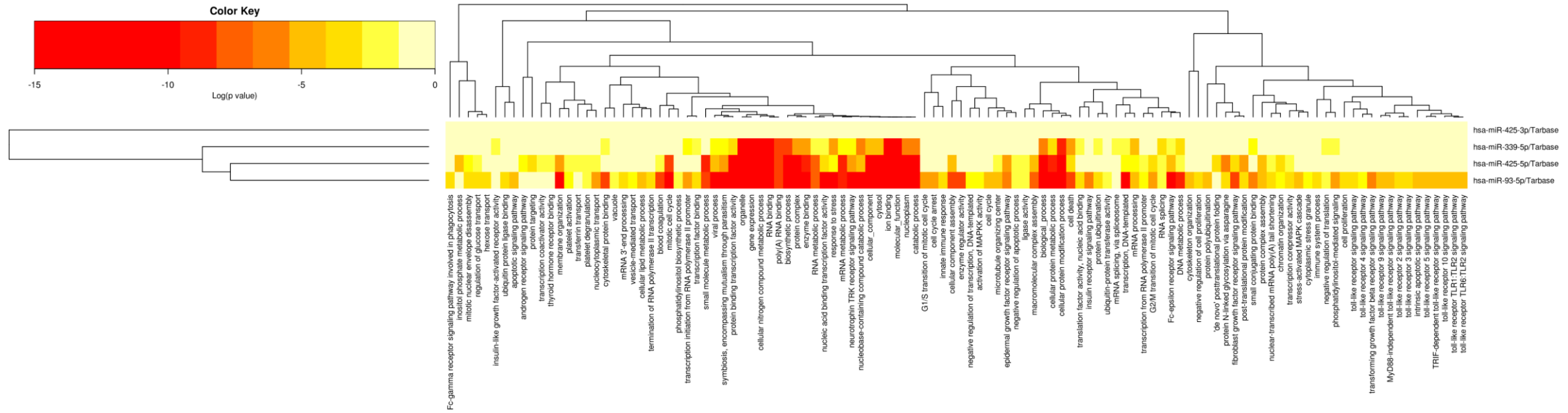

Supplement: Supplementary file 1 [file ijms-24-05081-s001.zip › Supplementary Figure S1.pdf]
